# Successful Treatment of Refractory Generalized Granuloma Annulare with Upadacitinib

**DOI:** 10.1155/2024/8859178

**Published:** 2024-06-05

**Authors:** Alexis Coican, Abigail Meckley, Nathan Sagasser, Melinda Greenfield, Eingun James Song, Jessica El-Bahri

**Affiliations:** ^1^Department of Graduate Medical Education, Orange Park Medical Center, Orange Park, FL 32073, USA; ^2^University of South Florida, 4202 E Fowler Ave, Tampa, FL 33620, USA; ^3^Advanced Dermatology and Cosmetic Surgery, 520 A1A N, Ponte Vedra Beach, FL 32082, USA; ^4^Frontier Dermatology, 3131 Nassau Street, Everett, WA 98201, USA

## Abstract

Granuloma annulare is a poorly understood dermatosis that, when generalized, can occur in up to 15 percent of patients. In these cases, treatment is frustrating and experimental. We report a case of a 60-year-old woman and a 41-year-old woman who demonstrated resolution of recalcitrant, generalized granuloma annulare (GA) following oral treatment with upadacitinib. After showing little to no response to other various treatments, such as steroids, antibiotic regimens, and systemic therapies, each patient was started on 15 mg of daily upadacitinib. At 2 months, one patient had complete clearance of all lesions while the other patient experienced noticeable improvement. Within 4 months, the other patient reached total resolution of her lesions. These cases provide evidence of a therapeutic option that may shorten disease duration and provide relief from cutaneous disease.

## 1. Introduction

Granuloma Annulare (GA) is a benign, cutaneous condition characterized by granulomatous inflammation of the dermis. GA presents as erythematous, annular plaques or papules and may be localized, disseminated, or subcutaneous. It is thought that GA is the result of a delayed-type hypersensitivity reaction, although the exact etiology of GA is unknown. However, the disease has been associated with thyroid disease, diabetes, certain infections (Epstein–Barr Virus, Varicella Zoster Virus, Human Immunodeficiency Virus, and tuberculosis), vaccinations, malignancy, and medications (TNF-α inhibitors, apremilast, and secukinumab) [1, 2]. Studies have shown upregulation of inflammatory pathways such as Th1 and Th2 in GA lesions compared to healthy skin. Additionally, expression of the Janus Kinase-signal transducer and activator of transcription (JAK-STAT) pathway, a driver of IFN-mediated inflammation, was also upregulated and implicated in the disease [3]. Although a multitude of treatment options exist, physicians lack a standardized, evidence-based treatment regimen for their patients with GA due to poor understanding of the pathogenesis of this disease. Without a better understanding of the disease, our patients are left with a condition that can persist for months to years. Recently, JAK inhibitor (JAKI) immunomodulation has gained popularity in treating cutaneous diseases including atopic dermatitis and psoriasis. Importantly, JAKIs have also shown promising effects when used off-label for other debilitating conditions such as alopecia areata, vitiligo, and even sarcoidosis [4]. Topical tofacitinib, a JAK1 and JAK3 inhibitor, has been reported to successfully treat both localized and generalized GA in a limited number of reports [4, 5]. To our knowledge, there are five reported cases of generalized GA responsive to upadacitinib, a selective JAK1 inhibitor [6–8]. Our case series documents two presentations of refractory disseminated GA that had complete resolution on treatment with upadacitinib.

## 2. Case Reports

### 2.1. Case 1

**Figure 1:**
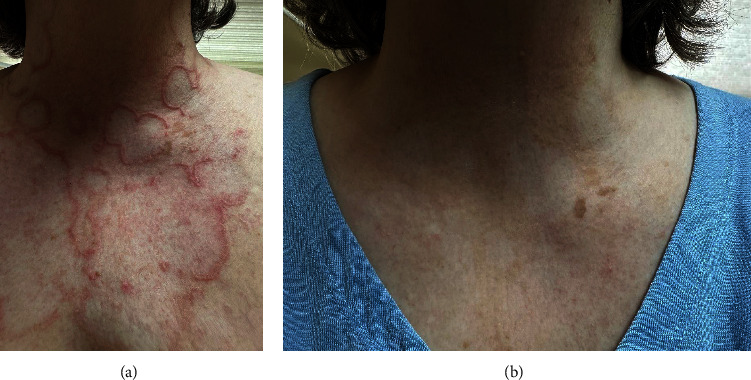
Chest before (a) and 4 months after (b) treatment with upadacitinib. Images demonstrate profound reduction in the erythematous, annular plaques.

**Figure 2:**
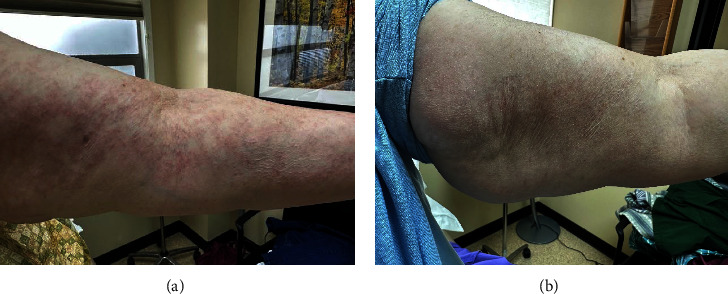
Left arm before (a) and 4 months after (b) treatment with upadacitinib. Images demonstrate resolution of pink to erythematous dermal papules and plaques.

A 60-year-old Caucasian female presented to the clinic with refractory GA for eleven years. Her past medical history includes hypothyroidism and diabetes mellitus type 2. At the time of presentation, the patient had inadequate responses to “ROM” therapy (rifampin, ofloxacin, and minocycline), ultrapotent topical steroids, dapsone, hydroxychloroquine, narrowband UVB, and pentoxifylline. The patient did achieve clearance with adalimumab; however, she had to switch treatment regimens due to lack of insurance coverage. Clinical examination revealed mildly pruritic pink to erythematous dermal papules and annular plaques involving the neck, chest, and diffusely throughout her back, inner arms, and inner thighs (Figures 1 and 2). The patient was agreeable to a trial of upadacitinib 15 mg once daily and all other treatments were discontinued. Eight weeks after the initiation of upadacitinib, clinical examination showed a noticeable improvement in redness and induration. Four months into treatment, her exam demonstrated complete clearance of all lesions with no adverse effects reported (Figures 1 and 2). The patient remains clear on 15 mg daily, six months later. If she continues to maintain clearance, attempts to taper off upadacitinib will be discussed after one-year total of daily therapy.

### 2.2. Case 2

**Figure 3:**
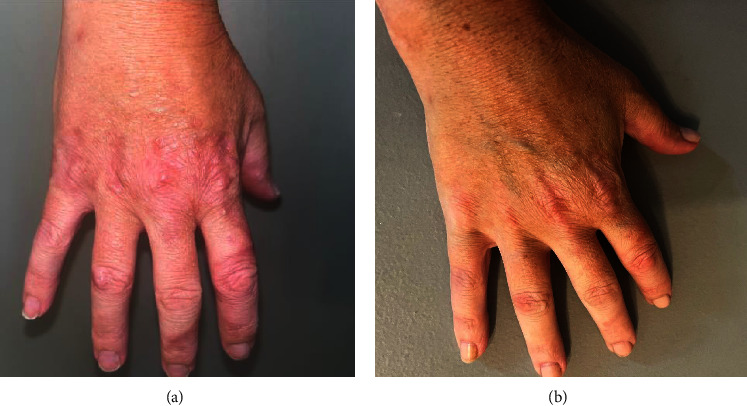
Right hand before (a) and after (b) treatment with upadacitinib for 2 months. Images demonstrate clearance of pink to erythematous dermal papules.

**Figure 4:**
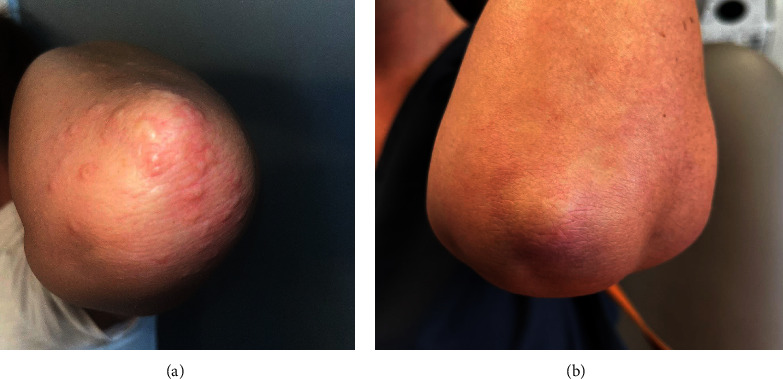
Left elbow before (a) and after (b) treatment with upadacitinib for 2 months. Images show resolution of pink dermal papules.

A 41-year-old female with a 21-year history of localized GA presented to the clinic with worsening, generalized GA. She had previously been treated with escalating potencies of topical steroids, oral steroids, methotrexate, hydroxychloroquine, low-dose naltrexone, and apremilast without significant improvement. In the three preceding months, she had been treated with apremilast with no improvement. On exam, she demonstrated mildly pruritic, erythematous, disseminated papules and nonscaly plaques around most of her joints, including bilateral knees, knuckles, and elbows (Figures 3 and 4). After discussion of treatment options, the patient elected to try a sample of oral upadacitinib 15 mg daily and discontinue other treatments. Follow-up evaluation at three weeks showed partial improvement in her condition, with 30% of the papules and plaques cleared and only mild headaches reported. At her two-month follow-up, the patient reached complete clearance of all lesions (Figures 3 and 4).

## 3. Discussion

Granuloma Annulare (GA) is a noninfectious, granulomatous disease with clinical variants including generalized or disseminated, perforating, patch, and subcutaneous. Localized GA is typically self-limiting and may resolve by itself or within months to a few years with traditional therapies; however, generalized granuloma annulare (GGA) may persist for decades and be unresponsive to traditional agents. Various therapeutic options exist for the treatment of GA, including corticosteroids, systemic therapies (methotrexate, hydroxychloroquine, apremilast, and TNF inhibitors), dapsone, pentoxifylline, and antimicrobials (rifampin, ofloxacin, and minocycline). In a systematic review of efficacious agents used to treat GGA, TNF-*α* inhibitors had the highest reported efficacy [9]. Interestingly, GGA has also been reported, although rarely, as an adverse class effect of TNF-*α* therapy in patients with rheumatoid arthritis (RA) that ceases once the medication is discontinued [10, 11]. We present two cases of refractory, disseminated GA to call attention to upadacitinib as a promising therapeutic avenue. To date, we know of only five GGA cases reporting the robust effects of upadacitinib. Slater et al. demonstrated a case of refractory disseminated GA, with concomitant diabetes and hypothyroidism, that completely resolved with upadacitinib treatment in just four weeks [6]. In a report by Sondermann et al., daily upadacitinib in a patient with diabetes and RA led to remission of both her RA and recalcitrant GA [7]. Zheng et al. recently documented a series of three cases of long-standing generalized GA responsive to and maintaining clearance with upadacitinib [8].

Although the exact etiology of GA is unknown, some clinical triggers and associations provide hints as to the mechanism behind the disease. As mentioned previously, GA lesions tend to have an upregulation of Th1 and Th2 pathways, as well as JAK-STAT. A recent study reported by Min et al. showed a 5400-fold increase in interleukin-4 (IL-4) in GA lesions compared to normal skin which inspired an investigation into the association of GA with atopic comorbidities [3]. Interestingly, patients with GA were significantly more likely to have experience with allergic rhinitis, asthma, and eczema [12]. Therefore, it could be suggested that the mechanistic culprit responsible for GA is Th2 dysregulation.

Having demonstrated complete clearance of GGA with upadacitinib, our case series provides further insight into this valuable therapeutic option. In Table 1, we summarize the case reports available to date, to our knowledge, using upadacitinib for the treatment of GGA. These four cases, including two from this paper, all reported near-complete or complete clearance of GGA lesions within 13 months.

While upadacitinib exhibits effects of a favorable treatment option, our series possesses fundamental limitations. A larger, more inclusive cohort of patients is required in order to validate the long-term efficacy and safety profile. Additionally, longer observational periods are essential to adequately study the permanency of results. As more GGA patients are treated with JAKI, we will gain insight into the disease course after treatment tapering in regard to flare-ups and JAKI tolerance. The two new cases reported here show a quick onset, successful treatment with upadacitinib in the setting of refractory, longstanding GGA, eleven years in case one and twenty-one years in case two. Our findings provide further support that JAKIs, specifically upadacitinib, show promise as a potential first-line option for disseminated GA given the positive responses and treatment toleration in reported cases.

## Figures and Tables

**Table 1:** Patients treated with upadacitinib for granuloma annulare.

Study	JAKI	Patient age, sex	Clinical GA type	Comorbidities	Avg. disease duration, years	Previous therapies	Adverse effects of JAKI	Time to complete or near-complete clearance	Previous JAKI used
Slater et al. [6]	Upadacitinib 15 mg daily	57, female	Generalized	T2DM, hypothyroidism	0.5	ROM, TCS, UVB, ILK, ruxolitinib 2% cream	None	1 month	Ruxolitinib

Sondermann et al. [7]	Upadacitinib 15 mg daily	61, female	Generalized	T2DM, RA	4	TCS, etanercept, OCS	None	4 months	

Zheng et al. [8]	Upadacitinib 30 mg daily (reduced to 15 mg maintenance)	52, female	Generalized		4	TCS, UVB, tacrolimus 0.1% ointment	Generalized weakness	8 months	
	Upadacitinib 30 mg (reduced to 15 mg maintenance)	60, female	Generalized		5	Dapsone, TCS	Abdominal cramps, URI	13 months	
	Upadacitinib 30 mg (reduced to 15 mg maintenance)	61, female	Generalized			TCS, methotrexate, UBV		2 months	

Case 1	Upadacitinib 15 mg daily	60, female	Generalized	T2DM, hypothyroidism	11	ROM, TCS, dapsone, HCQ, UVB, pentoxifylline	None	4 months	

Case 2	Upadacitinib 15 mg daily	41, female	Generalized		21	TCS, OCS, HCQ, methotrexate, apremilast, naltrexone	Headache, mild	2 months	

RA: rheumatoid arthritis; T2DM: type 2 diabetes mellitus; HTN: hypertension; ILK: intralesional triamcinolone; TCS: topical corticosteroids; OCS: oral corticosteroids; HCQ: hydroxychloroquine; “ROM” therapy: rifampin, ofloxacin, and minocycline; URI: upper respiratory illness.

## Data Availability

The data used to support the findings of this study are included within the article.
